# Analyzing Global Research Trends on Medical Resident Burnout and Physical Activity: A Bibliometric Analysis (2005–2025)

**DOI:** 10.3390/healthcare13192535

**Published:** 2025-10-07

**Authors:** Hamdi Henchiri, Amr Chaabeni, Ismail Dergaa, Halil İbrahim Ceylan, Valentina Stefanica, Wissem Dhahbi, Chayma Harrathi, Safa Abidi, Abdullah H. Allihebi, Anis Jellad, Fairouz Azaiez

**Affiliations:** 1 Higher Institute of Sport and Physical Education of Sfax, University of Sfax, Sfax 3000, Tunisia; hamdyhenchiri@gmail.com (H.H.); fairouz.kyranis@yahoo.com (F.A.); 2 Research Laboratory Education, Motricité, Sport et Santé (LR19JS01), Sfax 3000, Tunisia; 3 Occupational Medicine and Professional Pathologies Department, Gafsa Regional Hospital, Gafsa 2100, Tunisia; harrathi.chayma@gmail.com; 4 Department of Physical Medicine and Rehabilitation, Faculty of Medicine, University of Monastir, Monastir 5000, Tunisia; amrch97@gmail.com (A.C.); anisjellad@gmail.com (A.J.); 5 Research Laboratory of Technology and Medical Imaging—LR12ES06, Center for Musculoskeletal Biomechanics Research, Faculty of Medicine, University of Monastir, Monastir 5000, Tunisia; 6 Faculty of Medicine of Monastir, University of Monastir, Monastir 5000, Tunisia; abidisafa97@gmail.com; 7 High Institute of Sport and Physical Education of Ksar Said, University of Manouba, Mannouba 2010, Tunisia; phd.dergaa@gmail.com; 8 Physical Activity Research Unit, Sport and Health (UR18JS01), National Observatory of Sports, Tunis 1003, Tunisia; 9 Physical Education of Sports Teaching Department, Faculty of Sports Sciences, Atatürk University, Erzurum 25240, Türkiye; 10 Department of Physical Education and Sport, Faculty of Sciences, Physical Education and Informatics, National University of Science and Technology Politehnica Bucharest, Pitesti University Center, 060042 Pitesti, Romania; 11 Research Unit “Sport Sciences, Health and Movement”, High Institute of Sports and Physical Education of Kef, University of Jendouba, Kef 8100, Tunisia; wissem.dhahbi@gmail.com; 12 Training Department, Police College, Qatar Police Academy, Doha 7157, Qatar; 13 Department of Physical Education, College of Sport Sciences and Physical Activity, King Saud University, Riyadh 11451, Saudi Arabia; aalliheibi@ksu.edu.sa

**Keywords:** bibliometric analysis, burnout syndrome, exercise interventions, healthcare workforce, medical education, occupational stress, physical activity, resident physicians, wellness programs, workforce sustainability

## Abstract

**Background**: Medical resident burnout is a critical threat to healthcare workforce sustainability, with physical activity (PA) posited as a protective factor. This bibliometric analysis maps the global research landscape on this topic from 2005 to 2025. **Methods**: Systematic search of the Web of Science Core Collection identified 110 relevant English-language articles. Performance analysis and scientific mapping were conducted using R and VOSviewer. **Results:** The field saw an annual growth rate of 3.35%, with a peak of 16 publications in 2019. The United States was the dominant contributor, accounting for 68% of the total output. Analysis identified several major thematic areas, including stress and behavioral factors, occupational mental health, and institutional support mechanisms. The findings reveal a rapidly growing but geographically concentrated body of research, underscoring a significant gap in globally representative evidence. **Conclusions:** This analysis provides a foundational map for future research, underscoring the need for institutional wellness programs incorporating PA, international collaborative studies, and policy-level interventions. We conclude that integrating physical activity is not a luxury but a critical strategy for healthcare system sustainability.

## 1. Introduction

Healthcare systems worldwide recognize that maintaining a competent and resilient medical workforce represents one of the most critical challenges facing modern medicine [1,2]. Medical residents, who provide a substantial portion of direct patient care, occupy a particularly vulnerable position due to their dual role as learners and clinical practitioners, often operating under intense pressure that significantly impacts their psychological and physical well-being [3,4]. The prevalence of burnout among this population has reached alarming proportions, with profound implications for healthcare quality, patient safety, and workforce sustainability [1,5,6].

Burnout syndrome, characterized by emotional exhaustion, depersonalization, and diminished sense of personal accomplishment, represents a complex occupational phenomenon that extends far beyond individual resilience [4,7]. This concept is well-established in theoretical models, such as the Job Demands–Resources model, which posits that burnout results from a chronic imbalance between high job demands and limited resources [8]. This syndrome develops from the chronic experience of uncompensated stress where the perceived needs of the training environment (excessive workloads, unrealistic expectations) persistently outweigh the available resources and support systems [5,9,10]. The clinical manifestations of burnout encompass psychological symptoms, including depression and anxiety, physical symptoms such as chronic fatigue and sleep disturbances, and behavioral changes that compromise professional effectiveness [2,7,11]. These mechanisms create a self-reinforcing cycle where burnout symptoms impair coping capacity, leading to further stress accumulation and clinical deterioration [4,12,13,14].

Contemporary research has identified numerous critical gaps that limit our understanding of burnout prevention and management strategies among medical residents.

1—Significant methodological inconsistencies exist across studies examining burnout prevalence, with estimates ranging from 10.2% in anesthesiology to 89.5% in orthopedic surgery, reflecting variations in assessment tools, population characteristics, and definitional criteria [15,16,17,18,19].

2—Substantial underrepresentation of data from developing countries and diverse cultural contexts limits the generalizability of current interventions and prevention strategies [2,6].

3—Insufficient robust statistical analyses have been conducted to identify independent risk factors and protective elements that could inform targeted interventions [3,19].

4—Limited investigation has been undertaken regarding the relationship between specific types, intensities, and frequencies of physical activity and various dimensions of burnout among medical residents [20,21].

5—Inadequate attention has been devoted to examining how digital health technologies and innovative wellness platforms can be leveraged to deliver scalable physical activity interventions [22,23]. Sixth, insufficient research has explored the cost-effectiveness of physical activity-based wellness programs in medical education settings, limiting institutional adoption [1,24].

Bibliometric studies are statistical analyses of published research that assess the quantity, quality, and impact of scientific literature, highlighting relationships between authors, publications, and research areas [25,26,27]. By identifying the most influential papers and mapping trends over time, these studies allow researchers to understand the evolution of ideas and developments in epidemiology, treatment, and prevention [25,28].

This bibliometric study aimed to systematically analyze global research trends examining the relationship between physical activity and burnout among medical residents, map the intellectual structure of this research field, identify key contributors and collaborative networks, and highlight emerging research priorities for advancing evidence-based wellness interventions.

## 2. Materials and Methods

### 2.1. Study Design

This comprehensive bibliometric analysis employed a structured, multi-phase approach that combined quantitative performance indicators with network-based scientific mapping techniques. The methodology followed established protocols for systematic bibliometric research, incorporating both descriptive analysis of publication patterns and advanced visualization of intellectual structures within the research domain.

### 2.2. Data Source and Search Strategy

A systematic search was conducted using the Web of Science Core Collection database, selected for its comprehensive coverage, reliable citation indexing, and established utility in bibliometric analyses. The search was executed on June 1, 2025, using carefully constructed keyword combinations designed to capture the intersection of burnout, physical activity, and medical residency training.

The complete search strategy employed the following terms: TS = ((“burnout” OR “occupational stress” OR “emotional exhaustion” OR “professional fatigue” OR “work-related stress” OR “job burnout” OR “compassion fatigue” OR “depersonalization” OR “cynicism” OR “mental exhaustion”) AND (“physical activit*” OR “exercise” OR “sport*” OR “fitness” OR “physical training” OR “aerobic” OR “resistance training” OR “yoga” OR “workout” OR “leisure-time physical activit*” OR “physical exercise” OR “motor activit*”) AND (“medical resident*” OR “resident physician*” OR “resident doctor*” OR “physician in training” OR “doctor in training” OR “internship and residency” OR “postgraduate medical education” OR “clinical resident*” OR “house officer*” OR “trainee physician*”)).

Search parameters were restricted to title, abstract, and author keywords. The temporal scope encompassed publications from 1 January 2005, to 1 June 2025. Document types were limited to original research articles and review articles, with language restriction to English publications in peer-reviewed journals indexed in Web of Science.

### 2.3. Inclusion and Exclusion Criteria

The inclusion criteria comprised original studies and reviews addressing both physical activity (or related constructs such as exercise, sports, or fitness) and burnout among medical residents or during residency training programs, published between 2005 and 2025 in English-language peer-reviewed journals indexed in the Web of Science.

Exclusion criteria included studies focusing on non-medical trainees (such as nursing, dental, or other healthcare professions), articles unrelated to either physical activity or burnout, editorials, letters to the editor, commentaries, meeting abstracts, and non-peer-reviewed documents. After systematic screening and eligibility assessment, 110 articles were retained for comprehensive bibliometric analysis (Appendix A).

### 2.4. Data Extraction and Processing

All selected records were downloaded in plain text format, including complete bibliographic information and cited references. Metadata were imported into R software (version 4.5.0) using the bibliometrix package for quantitative analysis and into VOSviewer (version 1.6.20) for network visualization and scientific mapping.

### 2.5. Analytical Approach

Performance Analysis: Utilizing Bibliometrix, we analyzed annual scientific production patterns, identified the most productive journals and authors, examined author-level metrics and institutional affiliations, and mapped country-level contributions and international collaborations.

Scientific Mapping: Advanced bibliometric techniques were applied, including keyword co-occurrence analysis using author keywords to identify dominant research themes, author co-citation analysis to assess intellectual structure through the identification of influential authors, and bibliographic coupling analysis to identify thematically related document clusters based on shared reference patterns. Network visualizations were generated in VOSviewer. The association strength method was used for normalization, and clustering was performed using the default resolution parameter (1.00) and a minimum cluster size of 5 items. For keyword co-occurrence, only keywords with at least five occurrences were included. For co-citation analysis, authors with a minimum of 2 citations were considered, while in bibliographic coupling, documents with at least 10 shared references were mapped. Node size reflects the frequency of occurrence of each keyword. Node color indicates cluster membership, with items in the same cluster being closely related. Lines represent co-occurrence links, with thickness proportional to link strength.

## 3. Results

### 3.1. Bibliometric Overview

**Figure 1:**
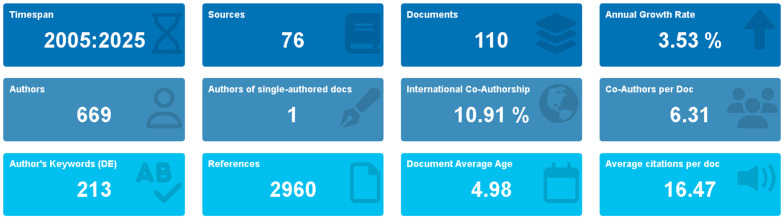
Main information. Note: The figure summarizes general characteristics of the studies; not all studies are listed in the reference list, only those cited in the text.

This comprehensive bibliometric analysis identified 110 publications examining the relationship between physical activity and burnout among medical residents, published across 76 distinct sources between 2005 and 2025. The research field demonstrated sustained growth with an annual growth rate of 3.35%. The collective impact of these publications was substantial, with an average of 16.47 citations per document (Figure 1 and Table 1).

### 3.2. Performance Analysis

#### Annual Scientific Production

**Figure 2:**
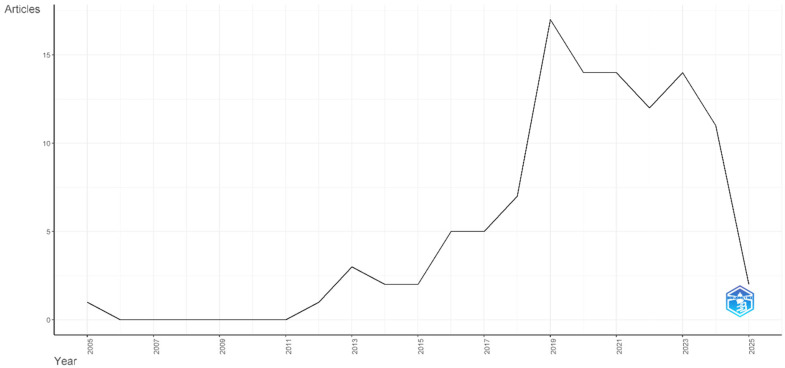
Annual production. Note: The blue symbol on the right is the R software signature, automatically added by the bibliometrix package used to generate this figure.

Analysis of temporal publication patterns revealed fluctuating but generally increasing productivity throughout the study period. The early years (2012–2018) showed modest but inconsistent output, followed by a dramatic acceleration beginning in 2019, which resulted in the highest annual production with 16 articles. This surge likely reflects increased awareness of physician burnout as a critical challenge for the healthcare system, potentially catalyzed by the growing recognition of workforce sustainability issues and heightened attention to the well-being of healthcare workers during the COVID-19 pandemic (Figure 2).

### 3.3. Most Productive Journals

**Figure 3:**
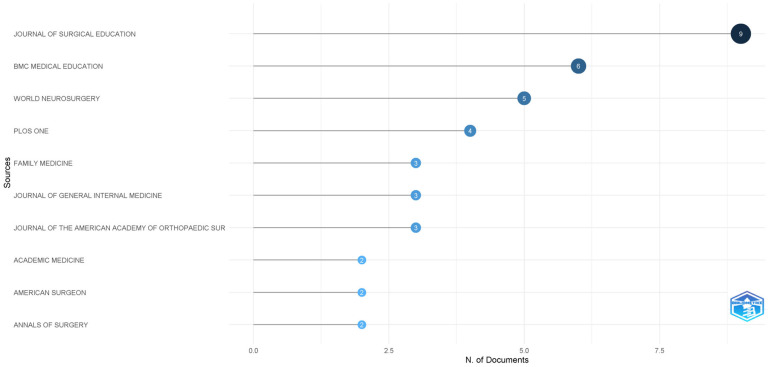
Most relevant journals.

The Journal of Surgical Education (impact factor 2.6) emerged as the leading publication venue, with nine articles, followed by BMC Medical Education (impact factor 2.7), which had six articles. This distribution highlights the particular relevance of burnout research within surgical training environments and broader medical education contexts, reflecting both the high-stress nature of surgical residency and the educational community’s commitment to trainee wellbeing (Figure 3).

### 3.4. Author Productivity and Impact

Spiotta, A.M., demonstrated the highest productivity with five publications, establishing consistent research engagement in this domain. However, Shanafelt, T.D. emerged as the most influential contributor with an H-index of 107 despite contributing only two articles, reflecting his foundational role in physician burnout research and the high impact of his scholarly contributions (Table 2).

### 3.5. Geographic Distribution and International Collaboration

**Figure 4:**
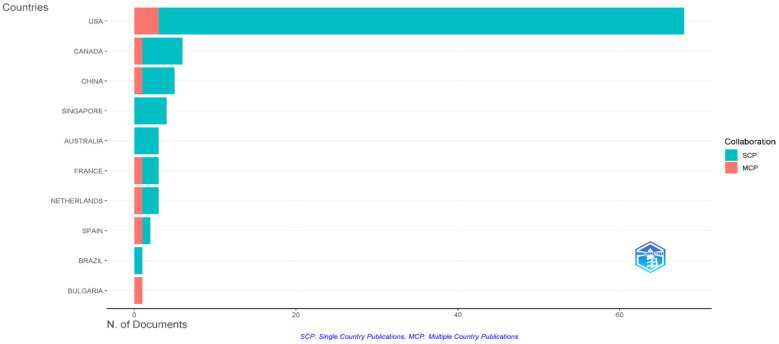
Corresponding authors’ countries.

The United States dominated the research landscape, accounting for 68 articles (68% of the total output), followed by Canada (5.5%) and China (4.5%). Authors from countries such as Singapore and Australia did not engage in international collaborations (Figure 4).

**Figure 5:**
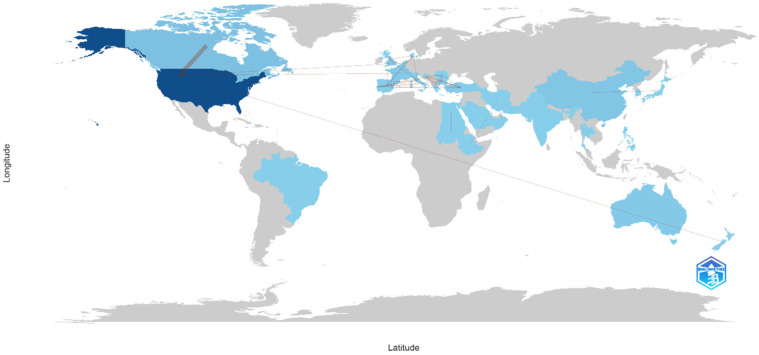
Countries collaborations. Each node represents a country, with node size and color intensity proportional to the number of publications. Darker blue shades indicate higher research productivity (e.g., the United States), while lighter blue shades represent countries with fewer publications. Lines connecting countries illustrate patterns of international collaboration based on co-authorship data.

The international collaboration network demonstrated the United States as the primary research hub, with substantial co-authorship connections to Canada, various European nations, and Australia, indicating established research partnerships and knowledge exchange networks. In contrast, African countries showed no collaboration with the United States and European countries (Figure 5).

### 3.6. Institutional Contributions

**Figure 6:**
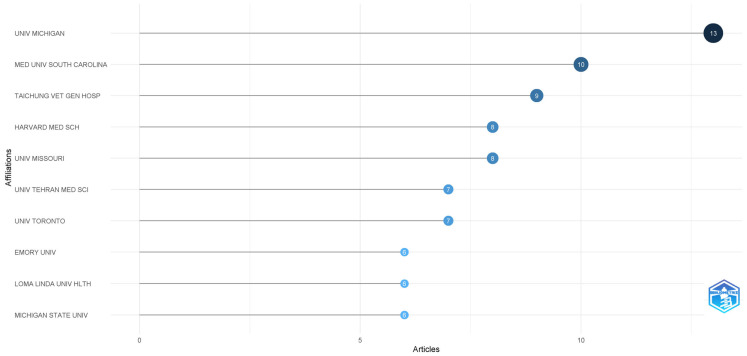
Most relevant affiliations.

The University of Michigan led institutional productivity with 13 articles, followed by the Medical University of South Carolina (10 articles), Taichung Veterans General Hospital (9 articles), Harvard Medical School (8 articles), and the University of Missouri (8 articles). This concentration reflects the research capacity and commitment of major academic medical centers to addressing workforce wellbeing challenges (Figure 6).

### 3.7. Scientific Mapping

#### Keyword Co-Occurrence Analysis

**Figure 7:**
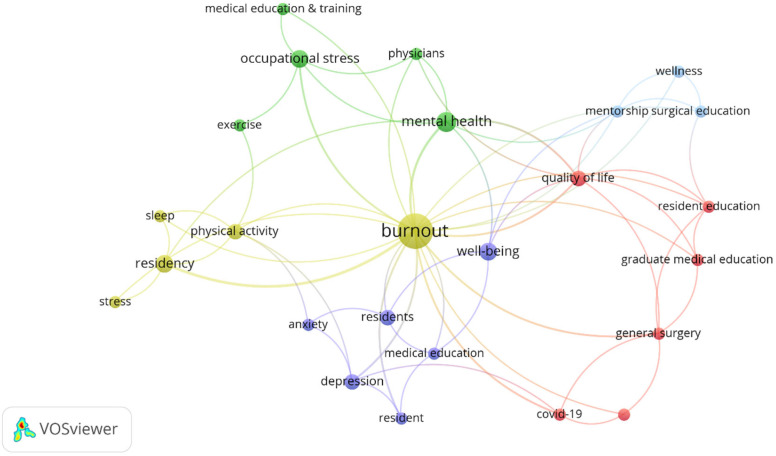
Keywords: co-occurrence network. Node size reflects the frequency of occurrence of each keyword. Node color indicates cluster membership, with items in the same cluster being closely related. Lines represent co-occurrence links, with thickness proportional to link strength.

The keyword co-occurrence network revealed five distinct thematic clusters centered around burnout as the core concept (Figure 7). These clusters encompassed: (1) stress and behavioral factors, prominently featuring physical activity and sleep as key variables; (2) occupational and mental health considerations; (3) pandemic-related educational disruptions and their impact on resident training; (4) psychological sequelae, including depression and anxiety; and (5) institutional support mechanisms such as mentorship programs and wellness initiatives. This thematic structure illustrates the multidimensional nature of burnout research and acknowledges physical activity as a key intervention strategy.

### 3.8. Author Co-Citation Analysis

**Figure 8:**
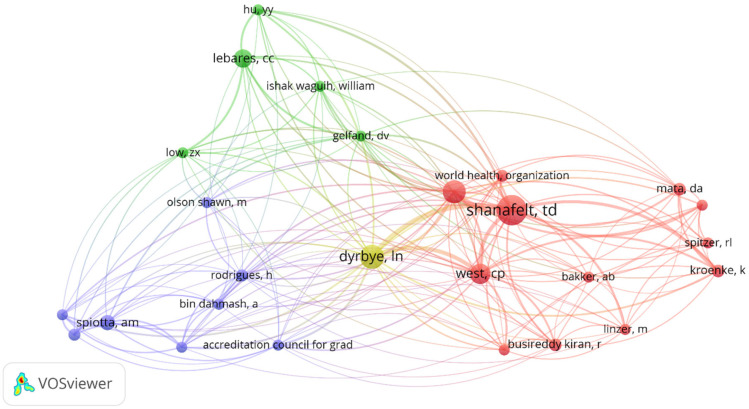
Authors’ co-citation network.

The author’s co-citation network identified several influential research clusters (Figure 8). The most prominent cluster centered around Shanafelt, West, and Dyrbye, representing the foundational community of physician well-being research. Additional influential clusters included Spiotta, Rodrigues, and Bin Dahmash (blue cluster), as well as Hu, Lebares, and Gelfand (green cluster), indicating diverse research approaches and collaborative networks within the field.

### 3.9. Bibliographic Coupling Analysis

**Figure 9:**
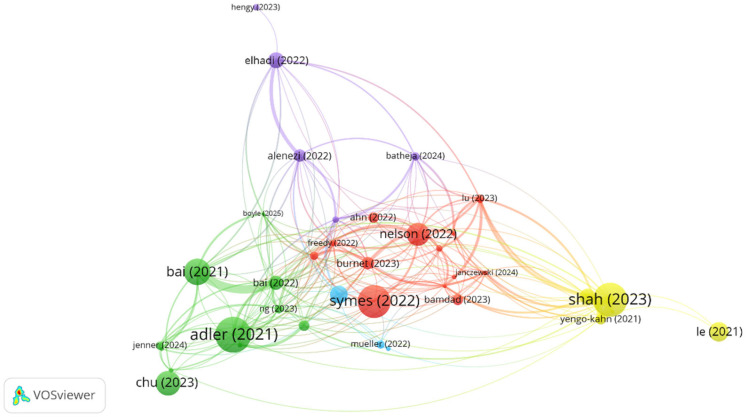
Bibliographic coupling network.

Bibliographic coupling analysis revealed five thematically distinct research clusters (Figure 9). The red cluster (“Coping strategies and wellness promotion”), including studies by Symes et al. (2022) [29], Nelson et al. (2022) [30], and Burnett et al. (2023) [31], focused on coping strategies, wellness promotion, and structural determinants of resident distress. The green cluster (“Digital health and behavioral risk profiling”), encompassing research by Adler et al. (2021) [32], Bai et al. (2021, 2022) [33], and Chu et al. [34], emphasized digital health approaches, behavioral risk profiling, and mental health considerations within predominantly Asian healthcare contexts. The yellow cluster (“Burnout in surgical specialties”) featured Shah et al. (2023) [35], whose systematic review comprehensively mapped burnout prevalence across surgical specialties, identifying modifiable factors such as working hours, autonomy levels, and wellness infrastructure. Peripheral clusters in blue and violet, including studies by Elhadi et al. (2022) [36], Alenezi et al. (2022) [37], and Maghbouli et al. (2021) [38], highlight the contextual constraints and resource limitations that drive burnout in low- and middle-income countries. The analysis highlights emerging lines of research, including organizational interventions, digital health approaches, and cross-specialty comparisons of burnout drivers. However, longitudinal designs, evidence from low- and middle-income countries, and the integration of physical activity into intervention frameworks remain underexplored.

## 4. Discussion

### 4.1. Global Research Trends and Knowledge Gaps

This bibliometric analysis documented a substantial geographic research concentration, with 68% of publications originating from the United States, directly confirming the identified gap in the introduction regarding the underrepresentation of diverse healthcare contexts [2,31]. The 3.35% annual growth rate peaked in 2019, coinciding with increased recognition of physician burnout as a healthcare crisis [1,29]. Network analysis revealed limited international collaboration, exceptionally minimal engagement between high-income and low- and middle-income countries, substantiating the introduction’s concern about insufficient global representativeness of current evidence [31,32].

### 4.2. Policy and Medical Education Implications

The observed annual growth rate of 3.35% and average citation rate of 16.47 per document demonstrate both increasing scholarly interest and substantial academic impact within this research domain [1,2]. The marked acceleration in publications since 2019 likely coincides with increased discussion about physician burnout as an urgent public health crisis that requires immediate attention from medical education institutions and healthcare systems worldwide [1,3,39,40]. This temporal pattern coincides with an increased awareness of healthcare workforce sustainability challenges, particularly following high-profile studies on physician burnout and growing evidence of its impact on patient safety and healthcare quality [9,10,39]. The relatively high citation suggests that the findings are being effectively translated into actionable interventions, demonstrating the practical relevance of this research domain for healthcare stakeholders worldwide.

The substantial geographic concentration, with the United States contributing 68% of publications, reveals significant research inequities that may limit the global applicability of current evidence [2,6]. This pattern reflects disparities in research infrastructure, funding availability, and academic publishing capacity between high-income and low- and middle-income countries [2,41]. Our analysis reveals that the majority of publications originate from the USA and other high-income countries. This focus may limit the generalizability of the findings and the applicability of proposed solutions in low- and middle-income regions, underscoring the need for more comprehensive geographical coverage in future studies. The documented geographic disparities in research output correspond with known variations in burnout prevalence: 28.1% for emotional exhaustion in low- and middle-income countries versus a pooled prevalence of 19.7% in European healthcare systems [41,42]. This evidence gap directly impacts the generalizability of interventions, addressing the need identified in the introduction for culturally adapted prevention strategies. The concentration of research within surgical education journals confirms specialty-specific vulnerability patterns, necessitating targeted policy responses within medical education frameworks [17,18]. This geographic bias represents a critical knowledge gap, as healthcare systems in resource-limited countries often face unique challenges, including inadequate staffing ratios, limited wellness infrastructure, cultural barriers to physical activity participation, and competing institutional priorities. The dominance of Western research perspectives may result in intervention strategies that are culturally inappropriate or resource-intensive for implementation in diverse global contexts.

### 4.3. Future Research Recommendations

#### 4.3.1. Thematic Evolution and Research Priorities

The identified thematic clusters translate into three concrete research priorities: (1) workplace intervention studies examining structured physical activity programs within residency curricula, (2) multi-institutional trials comparing burnout outcomes across training environments with varying wellness infrastructure, and (3) implementation science research evaluating scalable digital health platforms for burnout prevention [21,22,24]. The clustering patterns suggest that researchers are increasingly adopting biopsychosocial models that integrate neurobiological mechanisms (such as stress hormone regulation and neurotransmitter balance), psychological factors (including coping strategies and self-efficacy), and social determinants (such as workplace culture and peer support) in understanding the etiology of burnout and identifying intervention targets. The emergence of sleep as a co-occurring theme with physical activity indicates growing appreciation of the interconnected nature of lifestyle factors in burnout prevention, suggesting that effective interventions must address multiple behavioral domains simultaneously rather than targeting isolated risk factors.

The emergence of pandemic-related themes underscores the substantial impact of COVID-19 on medical education and resident well-being [3,40]. These disruptions highlighted existing vulnerabilities in residency training systems while accelerating interest in innovative wellness interventions, including digital health platforms and remote physical activity programs [21,22,23,24,41,42,43].

The pandemic fundamentally altered lifestyle behaviors, creating what researchers termed an “invisible pandemic of noncommunicable disease” due to reduced physical activity levels and increased sedentary behavior [44]. Studies from diverse global contexts demonstrated how physical activity behavior became a critical determinant of wellbeing, anxiety levels, and sleep quality during COVID-19 restrictions [45]. Research examining elite and sub-elite athletes during the pandemic showed long-term impacts on both mental health and nutritional practices, providing insights relevant to medical residents who face similar high-performance demands [46]. The sustained research interest in pandemic themes suggests that lessons learned during COVID-19 are being systematically incorporated into long-term wellness strategies, potentially improving healthcare system preparedness for future workforce challenges.

#### 4.3.2. Physical Activity as an Intervention Strategy

Research in this field has established preliminary evidence supporting the protective effects of physical activity against burnout, particularly in relation to emotional exhaustion and depersonalization [21,24]. Regular physical activity appears to maintain psychological well-being through multiple mechanisms, including sustained happiness, enhanced self-esteem, and increased optimism compared to sedentary counterparts [22]. However, conflicting findings regarding the consistency of physical activity-burnout relationships [47,48,49] highlight the need for more sophisticated research examining dose-response relationships, activity type specificity, and individual variation in responsiveness to interventions [50,51,52,53].

The bibliographic coupling analysis revealed diverse approaches to physical activity intervention, ranging from structured exercise programs to lifestyle modification strategies [21,22,24]. Digital health platforms emerge as promising tools for delivering scalable interventions, particularly given the time constraints and schedule unpredictability characteristic of residency training [22,23]. However, successful implementation requires careful attention to residency program culture, institutional support, and integration with existing wellness initiatives [1,24].

#### 4.3.3. Institutional and Policy Implications

Healthcare institutions require evidence-based implementation frameworks incorporating: mandatory protected time for physical activity (minimum 150 min weekly), structured mentorship programs with burnout screening protocols, and leadership accountability metrics for resident wellness outcomes [1,24]. Medical education accreditation bodies should mandate wellness competency assessments in conjunction with clinical performance evaluations [9,10]. Effective burnout prevention requires systemic changes, including duty hour optimization, autonomy enhancement, the establishment of mentorship programs, and the development of wellness infrastructure [1,5,54]. Physical activity interventions must be embedded within broader cultural changes that prioritize resident wellbeing as essential for healthcare quality and patient safety [9,10].

Residency programs require immediate policy reforms: duty hour caps with enforcement mechanisms, mandatory wellness curricula integrated into clinical rotations, and institutional wellness officer positions with direct reporting to program leadership [1,17,18].

#### 4.3.4. Future Research Directions and Innovation Opportunities

The identified thematic clusters indicate three methodological priorities addressing introduction gaps: (1) longitudinal randomized controlled trials examining dose-response relationships between physical activity modalities and burnout dimensions, resolving the identified gap in robust statistical analyses [21,24]; (2) multinational comparative effectiveness research incorporating diverse healthcare contexts, addressing geographic underrepresentation [41,43]; (3) cost-effectiveness analyses of digital health interventions, filling the identified void in economic evaluation research [22,24].

Expanding research to include diverse cultural contexts and healthcare systems is essential for developing globally applicable interventions [2,41]. Future studies should investigate how cultural attitudes toward physical activity, healthcare system structures, and resource availability influence the effectiveness and sustainability of interventions [41,42]. International collaborative research networks can facilitate knowledge exchange and the adaptation of interventions across diverse healthcare contexts [2,43].

### 4.4. Limitations

This bibliometric subject is subject to several methodological limitations that may impact interpretation. First, the exclusive use of the Web of Science database may introduce selection bias by omitting relevant studies published in regional journals or non-indexed sources, particularly affecting representation from low- and middle-income countries. Second, database-specific indexing procedures and citation counting methods may influence author impact assessments and topic trend identification. Third, bibliometric analysis provides quantitative patterns without qualitative evaluation of research methodology quality or intervention effectiveness. Citation-based indicators exhibit systematic bias toward senior researchers and established institutions, potentially misrepresenting emerging scholarly contributions and perpetuating existing academic hierarchies [26,27]. Fourth, the English-language restriction may exclude valuable research published in languages other than English, potentially affecting the global representativeness. Fifth, this study did not assess the methodological quality of the included publications, as bibliometric analysis focuses on publication patterns and citation metrics rather than evaluating the quality of individual studies. Internal validity threats warrant explicit acknowledgment. Omitted variable bias may influence findings through unmeasured institutional characteristics affecting both research productivity and citation impact, including funding availability, infrastructure quality, and academic prestige [25,26]. Confounding factors may potentially distort the observed associations between geographic location and research output, as resource-rich institutions may simultaneously produce higher publication volumes and attract greater citation rates, independent of research quality [27]. Reverse causality presents methodological concerns, whereby established researchers with existing citation networks may receive disproportionate recognition, regardless of the individual study’s merit, creating spurious associations between author productivity and scholarly impact [25]. Additionally, citation-based metrics may reflect academic networking patterns rather than scientific rigor, limiting the validity of impact assessments across different institutional contexts. Finally, the rapid evolution of this research field means that very recent publications may not yet have fully realized their citation potential, potentially underestimating their ultimate impact.

## 5. Conclusions

This comprehensive bibliometric analysis reveals an exponentially expanding research landscape, examining the relationship between physical activity and burnout among medical residents. This bibliometric analysis provides empirical documentation of research patterns that directly address identified knowledge gaps in the scholarship on medical resident burnout and physical activity. The systematic mapping of geographic inequities, methodological limitations, and intervention gaps establishes evidence-based priorities for allocating research funding and developing policy. Bibliometric analysis serves as an essential methodological tool for translating publication patterns into targeted wellness policy implementation within medical education systems.

## Figures and Tables

**Table 1:** Synopsis of key bibliometric findings.

Category	Metric	Value
**Publication Trends**	Total publications analyzed	110
	Temporal scope	2005–2025
	Annual growth rate	3.35%
	Peak publication year	2019 (16 articles)
	Average citations per document	16.47
**Geographic Distribution**	Leading country	USA (68.0%)
	Secondary contributors	Canada (5.5%); China (4.5%)
	International collaboration	Limited outside high-income countries
**Institutional Leadership**	Most productive institution	University of Michigan (13 articles)
	Secondary institutions	Medical University of South Carolina (10); Taichung Veterans General Hospital (9)
**Author Productivity**	Most prolific researcher	Spiotta, A.M. (5 publications)
	Most influential researcher	Shanafelt, T.D. (H-index: 107)
**Research Focus**	Primary thematic clusters	Stress management; occupational health; institutional support
	Emerging research areas	Digital health interventions; pandemic impacts

Percentages reflect proportional contribution to total publication output. The H-index represents the cumulative research impact across a career’s publications.

**Table 2:** Most productive authors.

Authors	Articles	H-Index
Spiotta, Alejandro M.	5	46.00
Fargen, Kyle M.	3	40.00
Patel, Sunil	3	25.00
Turner IV, Raymond D.	3	41.00
Shanafelt, Tait D.	2	107.00
Srijan Sen	2	37.00
Dyer, George Sinclair Mitchell	2	24.00
Chang, Qing	2	19.00
Morgan, Helen K.	2	16.00

## Data Availability

The authors will make the raw data supporting the conclusions of this article available upon request.
